# How does urbanization affect public health? New evidence from 175 countries worldwide

**DOI:** 10.3389/fpubh.2022.1096964

**Published:** 2023-01-06

**Authors:** Zhenhua Zhang, Mingcheng Zhao, Yunpeng Zhang, Yanchao Feng

**Affiliations:** ^1^Institute of Green Finance, Lanzhou University, Lanzhou, China; ^2^School of Management, Lanzhou University, Lanzhou, China; ^3^Business School, Zhengzhou University, Zhengzhou, China

**Keywords:** urbanization, public health, living standards, system GMM estimation, mechanism analysis

## Abstract

Urbanization is an essential indicator of contemporary society and a necessary historic stage in the industrialization of all countries. Thus, we explore the impact of urbanization on public health using the OLS estimation and a two-way fixed effect model based on annual panel data from 175 countries from 2000 to 2018. This paper also addresses potential endogeneity issues and identifies causal relationships using the coefficient stability tests, system GMM, and instrumental variable method. The results demonstrate that urbanization positively affects public health. Furthermore, we find that the impact of urbanization on public health can be mediated through living standards, and nations with higher living standards reduce the effect of urbanization on public health. An increase in the urbanization rate can promote public health by improving residents' living standards. Our results have significant real-world implications for the research of urbanization and the formulation of public health policy.

## 1. Introduction

Urbanization is the process of transforming rural population into an urban population (1), and is usually taken as a measure of social and economic development. The United Nations predicts that by 2050, the urban population will grow to 6.252 billion, with an urbanization rate of 67.2%. Rapid urbanization has given rise to “urban disease”, causing many social and environmental problems, such as, the disorderly development of urban space, excessive population aggregation, overemphasis on economic development, ignoring environmental protection, severe traffic congestion, shortage of energy resources, deterioration of the ecological environment, and thus on (2). How does urbanization affect public health? This is a question of great theoretical and practical significance. However, few studies focused on this issue in recent years, and the empirical evidence is relatively lacking.

Public health is one of the critical concerns of countries in the development process. The existing studies' ideas and methods of analyzing the relationship between urbanization and public health provide a solid reference for this paper. From the perspective of crude mortality and life expectancy at birth, this paper uses annual panel data of 175 countries from 2000 to 2018 to comprehensively evaluate the impact of urbanization on public health using multiple causal reasoning methods. We also adopt several kinds of robustness tests to increase the accurate of our findings.

The potential innovations of this paper are as follows: First, in the field of research data, we further expand the capacity of public health indicators and the number of countries and employs more extensive data to evaluate the relationship between urbanization and public health, making the evaluation effect more accurate. Most of the data in the existing literature cannot consider both time and region at the same time, and generally, there is a long-time span but only focus on a specific area, which is not comprehensive. For example, although Kegler et al. (3) obtained the relationship between urbanization and public health, its research scope only focused on the United States and was not universal. Although Li et al. (4) examined the relationship between the above two, they only used data from China. Such conclusions may not hold true on a global scale. Therefore, this paper uses panel data covering major countries in the world with a longer time span to conduct empirical research, which can more accurately assess the causal relationship between the two and obtain more general conclusions. It extends previous studies and enriches the literature in related fields.

Second, the relevant literature on the impact of urbanization on residents' health is not common, and most of it is a simple comparison of the health gap between urban and rural residents or a review (2), which lacks rigorous research based on empirical studies. In the existing empirical studies, some researchers adopted questionnaires for their analysis (5, 6). The lack of flexibility in the questionnaire will limit the responses of the respondents, and some more detailed and in-depth information may be omitted. Few studies based on econometric models mainly only conduct some simple regression and do not give clear answers in causal identification and robustness test of results (7, 8). This paper not only empirically tests the impact of urbanization on public health but also employs the bounding value analysis method of Oster (9), system GMM estimation, and the instrumental variable method of Lewbel (10) to determine the causal relationship between the two, and ensures the reliability of the results through a series of robustness tests. This study makes up for the lack of empirical evidence.

Third, living standards are closely related to urbanization and public health, and many studies have shown the pairwise links between the three (11, 12). In terms of channel analysis, this paper pioneeringly selects living standards as the mediating channel of urbanization rate affecting public health revealed the logic behind the mechanism of urbanization impact on public health for the first time. It can open the “black box” of the channel between urbanization impact and public health, and further strengthen the overall knowledge of the relationship between the two, enrich the relevant theories, and provide a more accurate reference for the government when formulating development policies.

The remaining parts of this paper are arranged as follows: the second part is the literature review; the third part is the research design and data situation; the fourth part is the empirical analysis, including the preliminary analysis and benchmark regression analysis; the fifth part is causal identification issues and strategies. The sixth part is a further discussion of the potential channel analysis; the seventh part summarizes the conclusion and proposes the policy suggestion. In Appendix, we carry out the robustness test.

## 2. Literature review

The impact of urbanization has long been a contentious issue. Researchers have noticed that increasing urbanization will have an impact on various aspects. For example, urbanization is closely related to economic development. Some scholars believe that the increase in urbanization rate can change the industrial structure, improve industrial production efficiency (13), enhance regional innovation ability, and drive the development of surrounding areas (14). Positive urbanization will promote the healthy development of the economy (15). However, the improvement of urbanization will accelerate the development of limited resources, contributing to many environmental pollution problems. Urbanization will worsen water quality (16) and increase carbon dioxide emissions (17). Urbanization changes the natural factors within the geographic system, and urban expansion and urban agglomeration may change the global distribution of PM_2.5_ concentration (18, 19), leading to a rise in PM_2.5_ concentration (20). In addition, the increase in urbanization rate also significantly impacts other aspects of development. The research of Satterthwaite et al. (21) proved that urban expansion would lead to the lack of agricultural land and the urban bias of infrastructure, services, and subsidized public funds.

For a long time, public health has been widely concerned by scholars. From the previous research content, the relevant research mainly focuses on two aspects. The first part mainly focuses on the influencing factors of public health, such as health expenditure (22), environmental policy (23), lifestyle, and social status (24). In addition, relevant studies have also shown that medical resource allocation (25), family-level harmony (26), and education (27) are all key influencing factors of public health. In the second aspect, scholars focus on selecting and constructing public health measurement indicators. Most of the public health indicators in existing studies are related to physical health. Examples include empirical stress and obesity rates (28), respiratory diseases (29), mortality (30), and life expectancy (31). Thurber et al. (32) adopted age-standardized health indicators, considering the correlation between physiological health indicators and age.

The impact of urbanization on public health has always been a controversial topic, and different scholars have different opinions on it. Some researchers argue that urbanization is harmful to public health through many channels. First, rapid urbanization will lead to a series of environmental pollution problems (33), including water pollution (16), dust pollution (34), and carbon dioxide emission (35) in urban residents' daily life. Environmental pollution is widely believed to harm citizens' health (36–39). Second, urbanization may lead to more chronic diseases and mental illnesses. Son et al. (40) adopted the Community Health Survey data in South Korea from 2008 to 2010 and found that urbanization would cause more asthma among residents. According to the research of Lambert et al. (41), urbanization will cause anxiety and emotional disorders, which is not conducive to the mental health of residents and will increase the incidence of infectious diseases (42, 43), affecting public health levels. Third, urbanization has changed people's living and working habits, resulting in adverse health effects. For example, Patil (44) found that urbanization changed diet and exercise habits, leading to the risk of obesity and overweight. Gong et al. (1) revealed that urbanization had led to changes in human activity patterns, diet, and social structure in China, resulting in frequent hypertension and other diseases. Fourth, in the process of urbanization, the supply of infrastructure and medical facilities commonly lags behind the speed of population agglomeration, which leads to various problems such as population crowding and difficult medical treatment (1, 45), which harms public health.

**Figure 1:**
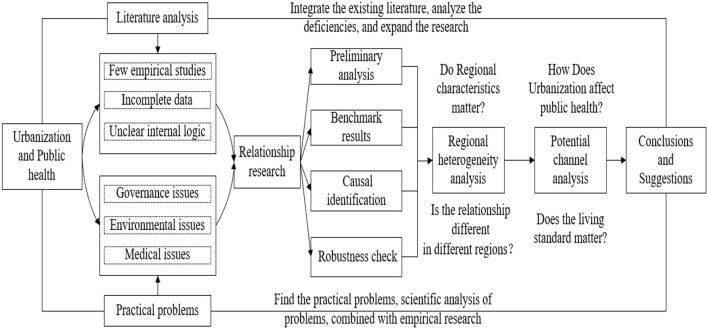
Research framework of the impact of urbanization on public health.

In contrast, another view holds that urbanization positively affects residents' health. The role of urbanization in promoting public health is mainly reflected in medical services. The level of medical and health services is compatible with urbanization development (46). There are significant differences in health resources between urban and rural areas (47). Health insurance coverage is higher in urban areas than in rural areas (48), and urban residents benefit from improved sanitation facilities (49). Urbanization also indirectly affects public health by affecting education and income. Lounkaew (50) used data from the PISA 2009 literacy test in Thailand and concluded that the education level of urban students was higher than that of rural students. As mentioned above, education is an important factor influencing public health (27). Chauvin et al. (51) compared urbanization in the United States, Brazil, China, and India and found that urbanization would lead to an urban-rural income gap, and India had the largest urban-rural income gap. Residents' income significantly affects their health level (52), while urbanization indirectly affects public health by increasing the income level of urban residents. Based on literature review and reality analysis, we propose a research framework (see Figure 1).

## 3. Research design

### 3.1. Econometrics model

In 1972, Grossman first analyzed residents' micro-health demand by establishing health production functions. This function considers many factors, including income pattern, living standards, education, environment, etc. (53). The model has been enriched and perfected in subsequent studies (54–56).

With the continuous increase of urban population and urbanization rate, the demand for public health services also increases. Urbanization has led to improvements in living standards, education and regional investment in public health (49, 50). On the contrary, regional environmental conditions, residents' working pressure and other factors may be negatively affected by urbanization (17, 44). Therefore, it is reasonable to take urbanization as an explanatory variable affecting residents' public health.

Based on the relevant literature and practical experience (57–60), real GDP per capita, primary school enrollment rate, women's fertility rate and domestic private health expenditure is closely related to public health and health conditions. Therefore, we take these factors as control variables to control the impact of these factors on public health status. According to the Grossman model, a simplified health production function can be expressed as:


(1) $$\begin{array}{l} H=G\left(X\right)=G\left(X_{1},X_{2},X_{3}...,X_{n}\right) \end{array}$$

where *H* represents the resident health variable and *X*_*i*_ (i = 1, 2, 3, … *n*) represents the factors affecting public health.

If the vector *X*_*i*_ affecting individuals is converted into a set of variables representing urbanization, economy, health expenditure, education, life standard, trade, foreign direct investment, the health production function can be expressed as C-D (Cobb- Douglas) production function:


(2) $$\begin{array}{l} H=G(Urb,Eco,Hex,Edu,Lst,Tra,FDI,O)\\ =\text{Ω}Urb_{i}{}^{\alpha_{i}}Eco_{i}{}^{\beta_{i}}Hex_{i}{}^{\gamma_{i}}Edu_{i}{}^{\lambda_{i}}Lst_{i}{}^{\eta_{i}}Tra_{i}{}^{\theta_{i}}FDI_{i}{}^{\sigma_{i}}O_{i}{}^{\mu_{i}} \end{array}$$

where (*Urb, Eco, Hex, Edu, Lst, Tra, FDI, O*) represents urbanization, economy, health expenditure, education, life standard, trade, foreign direct investment and other factors affecting health, respectively, α, β, γ, λ, η, θ, σ, μ is the corresponding elastic coefficient, and Ω is the estimated value of the initial public health. This paper selects relevant economic variables, health variables, education variables and life variables for empirical analysis. In addition, in the robustness test, this paper adds trade variables and FDI variables. By taking the logarithm Equation (2), Equation (3) is obtained:


(3) $$\begin{array}{l} \text{log}H=\text{logΩ}+\alpha_{i}\text{log}Urb_{i}+\beta_{i}\text{log}Eco_{i}+\gamma_{i}\text{log}Hex_{i}\;\\ +\lambda_{i}\text{log}Edu_{i}+\eta_{i}\text{log}Lst_{i}+\theta_{i}\text{log}Tra_{i}+\sigma_{i}\text{log}FDI_{i}+\mu_{i}\text{log}O_{i} \end{array}$$

Based on Equation (3), the econometric model of this paper is set as follows:


(4) $$\begin{array}{l} Health_{it}=\alpha_{0}+\alpha_{1}Urbanization_{it}+\alpha_{2}Control_{it}+u_{it} \end{array}$$

where *Health*_*it*_ refers to the public health condition of country *i* in year *t*, and *Urbanization*_*it*_ is the urbanization level of country *i* in year *t*. *Control*_*it*_ is a matrix of control variables to control for other factors that may impact public health. *u*_*it*_ is the stochastic disturbance term.

### 3.2. Variable selection and data source

Crude death rate and life expectancy at birth are two indicators used to measure public health in a specific region and are widely used by scholars (61, 62). Therefore, we select crude death rate(log*death*) and life expectancy at birth(log*life*) as dependent variables in this paper. For comparative study, we also subdivide life expectancy at birth into male life expectancy at birth(log*lifem*) and female life expectancy at birth(log*lifef*). These two variables are also added to the explained variables.

To measure the level of urbanization which is an explanatory variable, we refer to relevant literature (63, 64) to measure the urbanization level of a region by the urbanization rate. This paper measures the urbanization rate by the urban population ratio to each country's total population.

Our study uses annual panel data for 175 countries from 2000 to 2018 (list of countries in Appendix section), with data from the World Development Indicators (WDI) database in World Bank. The time period was chosen because the World Bank database lacked data before 2000, and urbanization in many developing countries had just started since 2002. According to the data of the World Bank, the growth rate of the global urbanization rate has been < 2% since 2018, significantly slowing down. Therefore, the sample time selected in this paper is from 2000 to 2018. We take the logarithm of all the variables selected to eliminate heteroscedasticity and reduce the amount of data for calculation. In addition, taking logarithms can also avoid interpretation difficulties due to inconsistent units. After taking the logarithm of the independent and dependent variables simultaneously, the estimated variable parameter can be interpreted as the elasticity.

### 3.3. Descriptive statistics

The descriptive statistics of the main variables are shown in Table 1. As can be seen from Table 1, the urbanization rate(*urban*) ranges from 8.246 to 100%, with a standard deviation of 22.97, indicating a considerable gap in the level of urbanization in various countries in the world. The mortality rate(*death*) is 8.461 deaths per 1,000 people and, even more alarmingly, 37.83 deaths per 1,000 live births. The average life expectancy(*life*) is 70.02 years, with a standard deviation of 9.123, and there are significant differences in life expectancy between sexes. More specifically, the average life expectancy for women(*lifef*) is 72.54 years and for men(*lifem*) is 67.59 years.

**Table 1:** Descriptive statistics.

	**(1)**	**(2)**	**(3)**	**(4)**	**(5)**
**Variables**	***N***	**Mean**	**SD**	**Min**	**Max**
*urban*	2,657	55.56	22.97	8.246	100
*gdp*	2,657	18,921	19,935	630.7	115,256
*school*	2,657	103.2	13.66	32.36	150.8
*fert*	2,657	2.900	1.517	0.977	7.679
*hexp*	2,657	41.11	18.66	1.196	86.44
*death*	2,657	8.461	3.342	1.127	20.43
*life*	2,656	70.02	9.123	42.52	85.42
*lifef*	2,656	72.54	9.550	44.60	86.80
*lifem*	2,656	67.59	8.859	40.42	84.10
*mort*	2,657	37.83	40.91	2	224.8
*mortf*	2,657	35.05	38.69	1.900	220.4
*mortm*	2,657	40.47	43.04	2.200	228.9
*clean*	2,377	62.94	38.07	0.290	100
*trade*	2,541	87.24	46.88	0.167	408.4
*fdi*	2,641	0.0585	0.184	−0.583	4.491
*elec*	2,657	78.74	30.66	1.243	100
*region*	2,657	2.809	1.559	0	5

## 4. Empirical results and discussion

### 4.1. Preliminary analysis

**Figure 2:**
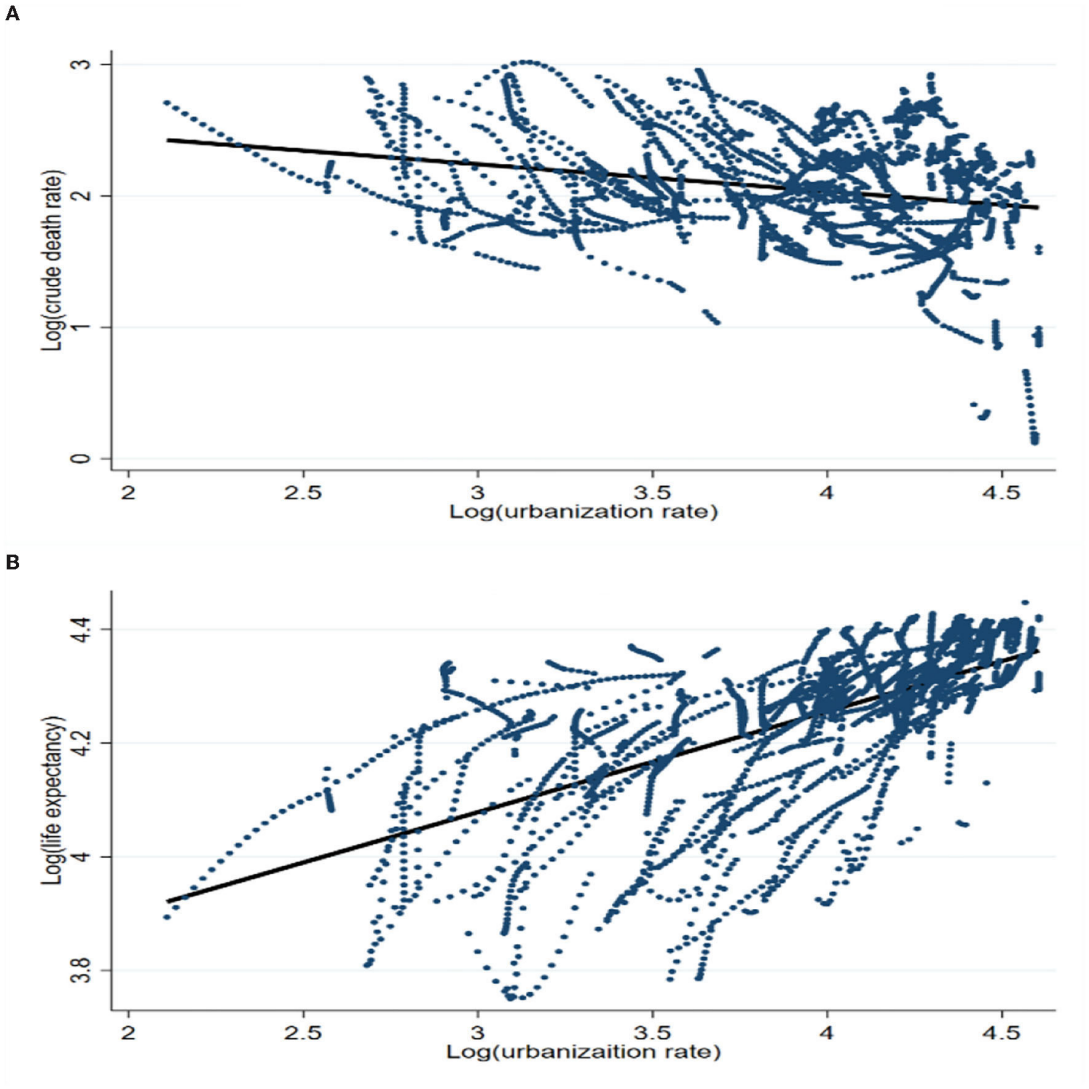
Health indicators and urbanization rate. **(A)** Crude death rate vs. urbanization rate. **(B)** Life expectancy vs. urbanization rate.

Before the formal regression begins, we plot scatter plots between the urbanization rate and the crude death rate and between the urbanization rate and life expectancy to visualize the relationship between the variables. As shown in Figure 2A, there is a negative correlation between the urbanization rate and the crude death rate. The result of linear fitting presents a linear relationship between them. As presented in Figure 2B, there is a high correlation between urbanization rate and life expectancy, and the relationship between them is linear.

Before regression analysis, we conduct a panel unit root test to avoid “spurious regression.” We performed unit root tests using the methods of PP-Fisher (65) and ADF-Fisher (66), which are unit root test methods specifically used for imbalanced panel data. Table 2 reports the results of the unit root test. As can be seen from the table, the null hypothesis of unit root is rejected for all variables, that is, they are stationary series.

**Table 2:** Panel unit root test results.

**Variables**	**Test**	
	**PP-Fisher**	**ADF-Fisher**
log*death*	74.2121^***^	47.0279^***^
log*urban*	140.6329^***^	94.6988^***^
log*gdp*	6.8772^***^	17.5851^***^
log*school*	4.1649^***^	22.052^***^
log*fert*	80.1766^***^	48.4945^***^
log*hexp*	15.1477^***^	26.0225^***^
log*life*	116.8599^***^	70.9718^***^
log*lifef*	135.0036^***^	78.4666^***^
log*lifem*	114.4151^***^	69.3512^***^
log*mort*	67.5314^***^	44.8545^***^
log*mortf*	62.6776^***^	43.3644^***^
log*mortm*	61.8747^***^	44.0279^***^
log*clean*	140.2575^***^	103.6408^***^
log*treat*	3.9968^***^	19.9974^***^
log*fdi*	38.5985^***^	44.507^***^
log*elec*	5.4126^***^	18.5051^***^

*** indicates significance at the 1% level.

### 4.2. Benchmark results

First, we estimate a linear regression model using the OLS estimation. Table 3 reports the OLS regression results of the impact of urbanization rate on public health. The empirical results in column (1) indicate that the urbanization rate has a strong negative effect on the crude death rate. More specifically, 1% increase in the urbanization rate reduces the crude death rate by 0.06%. The results in columns (2) to (4) show that the urbanization rate positively affects life expectancy at birth. Its coefficient is 0.014, which is statistically significant at the 1% level. While OLS estimation provide a simple answer, urbanization and public health indicators change over time and vary greatly across countries. The bias in coefficient estimation could be considerable, leading to misleading inference. As is shown in Table 4, we conduct four Hausman tests for four different dependent variables. The *p*-values in the statistical results are ~ 0, so the null hypothesis is rejected. Thus, we choose the fixed effects model. Considering the influence of the time factor, we use the two-way fixed effect model to carry out the analysis. Table 4 reports the results of the regression analysis of the two-way fixed effect model. The empirical results reveal that the coefficient of urbanization rate in column (1) is −0.653 at the 1% significance level. The results in columns (2) to (4) prove that a 1% increase in the urbanization rate leads to a significant 0.179% increase in life expectancy at birth. Regarding gender, urbanization increases women's life expectancy by 0.008% points more than men's.

**Table 3:** OLS estimation results.

	**(1)**	**(2)**	**(3)**	**(4)**
**Variables**	**log** ***death***	**log** ***life***	**log** ***lifef***	**log** ***lifem***
log*urban*	−0.058^**^	0.014^***^	0.018^***^	0.008
	(−2.39)	(2.91)	(4.00)	(1.64)
log*gdp*	−0.181^***^	0.048^***^	0.040^***^	0.057^***^
	(−13.05)	(17.68)	(15.54)	(19.63)
log*school*	−0.652^***^	0.069^***^	0.079^***^	0.059^***^
	(−12.30)	(6.71)	(8.03)	(5.31)
log*fert*	−0.348^***^	−0.119^***^	−0.143^***^	−0.093^***^
	(−13.06)	(−22.98)	(−28.82)	(−16.75)
log*hexp*	0.006	−0.013^***^	−0.014^***^	−0.012^***^
	(0.40)	(−4.79)	(−5.56)	(−4.10)
*Observations*	2,657	2,656	2,656	2,656
*R^2^*	0.166	0.714	0.746	0.670

^**^ and ^***^indicate significance at the 5 and 1% levels, respectively. Robust standard errors are in parentheses.

**Table 4:** Two-way fixed effect estimation results.

	**(1)**	**(2)**	**(3)**	**(4)**
**Variables**	**log*death***	**log*life***	**log*lifef***	**log*lifem***
log*urban*	−0.653^***^	0.179^***^	0.183^***^	0.175^***^
	(−3.79)	(4.06)	(4.22)	(3.87)
log*gdp*	−0.032	0.033^***^	0.035^***^	0.031^**^
	(−0.61)	(2.71)	(2.78)	(2.58)
log*school*	−0.349^***^	0.078^***^	0.079^***^	0.077^***^
	(−4.62)	(3.81)	(3.84)	(3.74)
log*fert*	0.227^**^	−0.037^*^	−0.049^**^	−0.023
	(2.60)	(−1.78)	(−2.41)	(−1.13)
log*hexp*	0.059	−0.015	−0.015	−0.014
	(1.55)	(−1.56)	(−1.60)	(−1.47)
*Constants*	Yes	Yes	Yes	Yes
*Country_FE*	Yes	Yes	Yes	Yes
*Time_FE*	Yes	Yes	Yes	Yes
*Observations*	2,657	2,656	2,656	2,656
*R^2^*	0.447	0.674	0.659	0.683

^*^, ^**^, ^***^ indicate significance at the 10, 5, and 1% levels, respectively; the values in parentheses of the regression coefficients are the standard errors of the clusters at the country level.

## 5. Causal identification issues and strategies

### 5.1. Excluding omitted variable bias

Before this part, we used a two-way fixed effect model for estimation. Although the two-way fixed effect model controls for country and year fixed effects and mitigates the omitted variable bias to some extent, the reality is highly complex, and many factors affect the independent and dependent variables. Therefore, the control of the two-way fixed effect model is limited. In particular, when factors that influence urbanization rates and public health and change over time are not fully taken into account, the resulting estimators are biased even using two-way fixed effect models. To solve the problem of inconsistent coefficient estimation, we refer to relevant literature and adopt the bounding value analysis of Oster (9).

Previous studies often carried out a coefficient sensitivity analysis by adding control variables. In the past, scholars believed that if the treatment effect coefficient was still stable when new observable variables were added to the model, the deviation caused by omitted variables would be considered small. However, these studies have ignored the information reflected by *R*^2^. Therefore, Oster (9) based on the hypothesis that the relationship between treatment effect and unobservable variables can be deduced from the relationship between treatment effect and observable variables and improved the robustness test method of omitted variable bias. When there are unobservable variables, the estimator $\beta^{*}=\tilde{\beta }-\left[\delta \left(\overset{•}{\beta }-\tilde{\beta }\right)\left(R_{\max}-\tilde{R}\right)/\left(\tilde{R}-\overset{•}{R}\right)\right]$ can be used to test whether the benchmark regression results are affected by omitted variables. $\overset{•}{\beta }$, $\overset{•}{R}$ and $\tilde{\beta }$, $\tilde{R}$ correspond to the estimated coefficients of the core explanatory variables and the goodness-of-fit of the regression equations when the constrained control variables and the observable control variables are introduced into the estimation, respectively. *R*_max_ represents the maximum goodness of fit when the unobservable variable can be observed. δ represents the relative strength of the correlation between observable variables and unobservable variables and the variables of concern.

We conduct a bounding value analysis of four health indicators used in this study. We set *R*_max_ to 1.3 $\tilde{R}$ and δ to 1. The results of the bound estimation are presented in Table 5. To make the results more precise, we reproduce the above regression results using two-way fixed effect estimation in column (1) of Table 5. Column (2) of Table 5 reports the bound estimation. The result shows that the interval formed by β^*^ and $\tilde{\beta }$ does not contain 0. It means that our two-way fixed effect estimations are robust to potential omitted variable bias. In addition, by comparing columns (1) and (2), we find that the coefficient of the impact of the urbanization rate on the crude death rate changes from −1.128 to −0.653 after considering the omitted variable bias. This indicates that the impact of the urbanization rate on the crude death rate is stronger after accounting for omitted variable bias. We find the same results for other health measures.

**Table 5:** Bound estimation results.

	**(1) Controlled effect**	**(2) Identified set**
	$\hat{\beta }\left(S.E.\right)$	$\left[\hat{\beta },\beta^{*}\left(Min\left\{1,1.3{\hat{R}}^{2}\right\},\delta =1\right)\right]$
log*death*		
log*urban*	−0.653^***^ (−3.79)	[−1.128, −0.653]
*Observations*	2,657	
*R^2^*	0.447	
log*life*		
log*urban*	0.179^***^ (4.06)	[0.179, 0.457]
*Observations*	2,656	
*R^2^*	0.674	
log*lifef*		
log*urban*	0.183^***^ (4.22)	[0.183, 0.450]
*Observations*	2,656	
*R^2^*	0.659	
log*lifem*		
log*urban*	0.175^***^ (3.87)	[0.175, 0.463]
*Observations*	2,656	
*R^2^*	0.683	

^*^, ^**^, ^***^ indicate significance at the 10, 5, and 1% levels, respectively; the values in parentheses of the regression coefficients are the standard errors of the clusters at the country level.

### 5.2. System GMM estimation

To solve the problems of endogeneity and estimation bias that may exist in the model, we use the system GMM method for estimation. We also use two test methods that Arellano and Bover (67) proposed to verify the validity of instrumental variables and the system GMM estimation results. The first is the second-order serial correlation test AR (2), whose main function is to test whether the residuals estimated by the system GMM have a serial correlation. The second is the overidentification constraint test, which is mainly used to verify whether the instrumental variables used in the system GMM estimation are jointly effective. In the empirical study, the Hansen test method is adopted for identification.

Table 6 reports the test results of the system GMM model with crude death rate, life expectancy, female life expectancy, and male life expectancy as explained variables in turn. The *p*-values of the four second-order serial autocorrelation tests AR(2) are all >0.1, and the Hansen test is also >0.1, which means that there is no second-order serial correlation in the regression equation. The instrumental variables are generally valid. This confirms that the GMM model setup is reasonable. As shown in the table, urbanization negatively affects mortality and positively affects life expectancy. The positive impact of urbanization on public health is robust.

**Table 6:** System GMM estimation results.

	**(1)**	**(2)**	**(3)**	**(4)**
**Variables**	**log** ***death***	**log** ***life***	**log** ***lifef***	**log** ***lifem***
log*urban*	−0.024	0.001	0.005	0.004
	(−0.42)	(0.14)	(0.84)	(0.90)
log*gdp*	−0.239^**^	−0.022^*^	0.036	−0.059^**^
	(−2.28)	(−1.75)	(1.63)	(−2.44)
log*school*	0.297^*^	−0.001	−0.072^*^	−0.017
	(1.66)	(−0.04)	(−1.70)	(−0.42)
log*fert*	0.003	−0.013^***^	−0.010	−0.016^**^
	(0.01)	(−3.21)	(−1.58)	(−2.08)
log*hexp*	0.039	−0.002	−0.007	−0.006
	(1.36)	(−0.61)	(−1.13)	(−0.92)
*Constant*	Yes	Yes	Yes	Yes
*Country FE*	Yes	Yes	Yes	Yes
*Time FE*	Yes	Yes	Yes	Yes
*AR (2) p*-value	0.992	0.192	0.743	0.264
*Hansen test p*-value	0.918	0.708	0.532	0.883
*Observations*	2,141	2,141	2,141	2,141

^*^, ^**^, ^***^ indicate significance at the 10, 5, and 1% levels, respectively; the values in parentheses of the regression coefficients are the standard errors of the clusters at the country level.

### 5.3. 2SLS estimation

In addition, to make the results of this paper convincing, we further employ the instrumental variable method to conduct two-stage least squares regression to address the potential endogeneity problem. This paper employs the urbanization level of one-stage lag as an instrumental variable. We first take urbanization as the dependent variable, and the urbanization lagging behind by one period as the independent variable for regression to obtain the residual term. The residuals (log*urbanhat*) were used as explanatory variables to replace urbanization in model (1) for regression, and the results are shown in Table 7. Urbanization has significantly improved public health. Specifically, every 1% increase in the urbanization rate will reduce the crude death rate by 0.494%. The remaining coefficients are as expected. This proves that our results are reliable.

**Table 7:** 2SLS estimation results.

	**(1)**	**(2)**	**(3)**	**(4)**
**Variables**	**log*death***	**log*life***	**log*lifef***	**log*lifem***
log*urbanhat*	−0.494^***^	0.117^***^	0.113^***^	0.121^***^
	(−3.51)	(3.85)	(3.87)	(3.81)
log*gdp*	−0.002	0.037^***^	0.037^***^	0.037^***^
	(−0.06)	(4.53)	(4.59)	(4.34)
log*fert*	0.183^**^	−0.054^***^	−0.069^***^	−0.039^**^
	(2.32)	(−3.13)	(−4.06)	(−2.16)
log*hexp*	0.069^*^	−0.020^**^	−0.020^**^	−0.018^**^
	(1.77)	(−2.16)	(−2.24)	(−2.04)
log*school*	−0.365^***^	0.078^***^	0.080^***^	0.076^***^
	(−4.75)	(3.96)	(4.04)	(3.82)
*Constant*	Yes	Yes	Yes	Yes
*Country FE*	Yes	Yes	Yes	Yes
*Time FE*	Yes	Yes	Yes	Yes
*Observations*	2,377	2,377	2,377	2,377

^*^, ^**^, ^***^ indicate significance at the 10, 5, and 1% levels, respectively; the values in parentheses of the regression coefficients are the standard errors of the clusters at the country level.

### 5.4. Instrumental variable method

To further solve the possible endogeneity problem, we also construct a new and effective instrumental variable for re-estimation with the help of the heteroscedasticity instrumental variable method proposed by Lewbel (10). He introduced a new method that an instrumental variable could be constructed with the help of a set of observable exogenous variable vectors Z in the absence of relevant traditional instrumental variables or in the presence of weak instrumental variables.

The operation method is as follows: in the first stage, the endogenous variable is regressive to the exogenous variable Z, the residual term ε_2_ is obtained, and $\left(Z-\bar{Z}\right)\hat{\varepsilon_{2}}$ is constructed as the instrumental variable in the second step estimation, where $\bar{Z}$ is the mean value of the exogenous variable vector. This heteroscedasticity-based discrimination method requires that the residuals of the first stage regression be heteroscedasticity. In the second stage, the instrumental variables estimate in the first step are used to estimate the effect of the explanatory variables on the explained variables. According to Lewbel (10), this paper sets the following model.


(5) $$\begin{array}{l} Y_{1}=\beta X+\gamma Y_{2}+\varepsilon_{1} \end{array}$$

(6) $$\begin{array}{l} Y_{2}=\alpha X+\varepsilon_{2} \end{array}$$

where *Y*_1_ is the four indicators to measure public health, *Y*_2_ is the urbanization rate, *X* is all the control variables, and ε_1_ and ε_2_ represent the error terms. *Z*ϵ*X* or *Z* = *X*. We first estimate the residual term of Equation (6) by regression with the whole sample, and test the heteroscedasticity of the residual term. The null hypothesis of homoscedasticity can be rejected if the *p*-value is 0, and the existence of heteroscedasticity of the residual term of Equation (6) is proved. Then, the instrumental variables are constructed according to $\left(Z-\bar{Z}\right)\hat{\varepsilon_{2}}$, and Equation (5) is re-estimated. The results are listed in Table 8.

**Table 8:** IV estimation results.

	**(1)**	**(2)**	**(3)**	**(4)**
**Variables**	**log** ***death***	**log** ***life***	**log** ***lifef***	**log** ***lifem***
log*urban*	−2.171^***^	0.514^***^	0.519^***^	0.505^***^
	(−8.45)	(7.34)	(7.74)	(6.93)
log*gdp*	−0.027	0.034^***^	0.036^***^	0.032^***^
	(−0.81)	(4.44)	(4.51)	(4.13)
log*school*	0.026	−0.008	−0.007	−0.008
	(0.39)	(−0.41)	(−0.41)	(−0.37)
log*fert*	8.691^***^	−2.044^***^	−2.078^***^	−1.996^***^
	(8.76)	(−7.31)	(−7.73)	(−6.88)
log*hexp*	0.037	−0.011	−0.011	−0.010
	(1.13)	(−1.30)	(−1.33)	(−1.22)
*Constants*	Yes	Yes	Yes	Yes
*Country FE*	Yes	Yes	Yes	Yes
*Time FE*	Yes	Yes	Yes	Yes
*Observations*	2,657	2,656	2,656	2,656
*R* ^2^	0.616	0.745	0.734	0.750

^*^, ^**^, ^***^ indicate significance at the 10, 5, and 1% levels, respectively; the values in parentheses of the regression coefficients are the standard errors of the clusters at the country level.

The regression results of instrumental variables in Table 8 illustrate that urbanization significantly negatively impacts the crude death rate. Specifically, the coefficient of the urbanization rate is −2.171. Columns (2) to (5) of the table indicate that the level of urbanization significantly boosts life expectancy.

In summary, we try to solve the possible endogeneity problem of benchmark regression using Oster (9) bound estimation, system GMM estimation, and instrumental variable method. The results from Tables 5–8 all reveal that the urbanization rate significantly improves the public health level.

## 6. What is driving the results?

A key question is raised: What are the potential channels through which urbanization affect public health? This paper attempts to explore whether living standards can be such a channel. Related studies (12, 68) found a correlation between urbanization rate and living standards. In addition, it is widely believed that rising living standards improve public health. Real GDP per capita is commonly used to represent the standard of living (69, 70). For living standards to be a potential channel, two conditions must be met. First, real GDP per capita needs to be correlated with urbanization rates. Table 9 reports the relationship between the urbanization rate and real GDP per capita(log*gdp*). The results show that the increase in urbanization rate can significantly improve living standards. Specifically, for every 1% increase in the urbanization rate, the real GDP per capita increases by 0.573%.

**Table 9:** Effect of urbanization on the potential channel.

	**(1)**
**Variables**	**log** ***gdp***
log*urban*	0.573^***^
	(3.29)
*Controls*	Yes
*Constants*	Yes
*Country FE*	Yes
*Time FE*	Yes
*Observations*	2,657
*R* ^2^	0.599

^*^, ^**^, ^***^ indicate significance at the 10, 5, and 1% levels, respectively; the values in parentheses of the regression coefficients are the standard errors of the clusters at the country level.

Second, put living standards, urbanization rate, and public health into the same regression model, and the coefficient of urbanization rate should be reduced or insignificant. The results are presented in Table 10. Columns (2) and (5) of Tables 10, 11 show that when living standards, urbanization rate, and public health are added to the same regression model, the coefficient on urbanization rate decreases significantly. Our research suggests that the standard of living is one channel through which urbanization rates affect public health. We also examine whether living standards moderated the relationship between urbanization and public health to further probe its channel role. More precisely, we include an interaction term between urbanization rate and living standards (logurban^*^log*gdp*) in Equation (4), as shown in columns (3) and (6) of Tables 10, 11. The coefficient of the interaction term is significant and opposite to the coefficient of the urbanization rate. It means that living standards negatively moderate the impact of the urbanization rate on public health. In other words, the relationship between urbanization and public health is lower in countries with higher living standards.

**Table 10:** Test results of potential channel mechanism (crude death rate and total life expectancy).

	**(1)**	**(2)**	**(3)**	**(4)**	**(5)**	**(6)**
**Variables**	**log*death***	**log*death***	**log*death***	**log*life***	**log*life***	**log*life***
log*urban*	−0.672^***^	−0.653^***^	−0.305^*^	0.198^***^	0.179^***^	0.090^*^
	(−4.00)	(−3.79)	(−1.66)	(4.68)	(4.06)	(1.89)
log*gdp*		−0.032	0.032		0.033^***^	0.017^*^
		(−0.61)	(0.75)		(2.71)	(1.78)
logurban^*^log*gdp*			0.322^***^			−0.082^***^
			(4.29)			(−4.05)
*Controls*	Yes	Yes	Yes	Yes	Yes	Yes
*Constant*	Yes	Yes	Yes	Yes	Yes	Yes
*Country FE*	Yes	Yes	Yes	Yes	Yes	Yes
*Time FE*	Yes	Yes	Yes	Yes	Yes	Yes
*Observations*	2,657	2,657	2,657	2,656	2,656	2,656
*R* ^2^	0.446	0.447	0.520	0.665	0.674	0.714

^*^, ^**^, ^***^ indicate significance at the 10, 5, and 1% levels, respectively; the values in parentheses of the regression coefficients are the standard errors of the clusters at the country level.

**Table 11:** Test results of potential channel mechanism (female and male life expectancy).

	**(1)**	**(2)**	**(3)**	**(4)**	**(5)**	**(6)**
**Variables**	**log*lifef***	**log*lifef***	**log*lifef***	**log*lifem***	**log*lifem***	**log*lifem***
log*urban*	0.203^***^	0.183^***^	0.095^*^	0.193^***^	0.175^***^	0.086^*^
	(4.91)	(4.22)	(1.94)	(4.40)	(3.87)	(1.85)
log*gdp*		0.035^***^	0.019^*^		0.031^**^	0.015
		(2.78)	(1.97)		(2.58)	(1.53)
logurban^*^log*gdp*			−0.081^***^			−0.083^***^
			(−3.99)			(−3.99)
*Controls*	Yes	Yes	Yes	Yes	Yes	Yes
*Constant*	Yes	Yes	Yes	Yes	Yes	Yes
*Country FE*	Yes	Yes	Yes	Yes	Yes	Yes
*Time FE*	Yes	Yes	Yes	Yes	Yes	Yes
*Observations*	2,656	2,656	2,656	2,656	2,656	2,656
*R* ^2^	0.649	0.659	0.699	0.675	0.683	0.723

^*^, ^**^, ^***^ indicate significance at the 10, 5, and 1% levels, respectively; the values in parentheses of the regression coefficients are the standard errors of the clusters at the country level.

## 7. Conclusions and suggestions

Public health is a significant development goal and one of the priorities of government work all over the world. The concentration of population into cities and towns, known as urbanization, is a worldwide trend. And the process of urbanization itself is a process of economic development and modernization. This paper uses the annual panel data published by the World Bank from 2000 to 2018 to examine whether urbanization affects public health and potential channels.

First, increased urbanization has significantly improved public health. Urbanization has considerably reduced the crude death rate and increased life expectancy at birth. This further confirms the conclusions of Shen et al. (46) and Jiang et al. (56). From a gender-specific perspective, urbanization increases women's life expectancy at birth more than men's.

Second, the cause-and-effect relationship between increasing urbanization rate and improved public health status is valid. To solve the problem of causal identification and ensure the establishment of a causal mechanism of action, we use the bounding value analysis method, system GMM estimation, and the instrumental variable method to deal with the possible endogeneity problem. The results of these tests suggest that there is indeed a causal link between urbanization and public health.

Third, the results of our estimation are robust. We also perform robustness checks by replacing the explained and core explanatory variables, grouping regressions, and adding more control variables. The results demonstrate that our conclusion is credible.

Fourth, further research shows that the impact of urbanization on public health can be mediated through living standards. Specifically, the increase in urbanization rate significantly promotes residents' living standards, and the rise of residents' living standards positively impacts public health. In addition, living standards can also be used as a moderating variable of urbanization and public health. The impact of urbanization on public health is stronger in countries with low living standards than in countries with high living standards.

The results of our research presented above can provide some references for relevant policymakers. On one hand, policymakers should make improving the quality of urbanization development a top priority and strive to play a positive role and reduce its negative impact. On the other hand, the impact of urbanization on public health is stronger in countries with lower living standards. This conclusion has important implications for developing countries. In the context of rapid urbanization, policymakers in developing countries can further improve the level of health security of residents by promoting their urbanization.

There are still some limitations in this paper. First, in terms of the potential channel of urbanization affecting public health, only living standards is selected as the mediating variable. Future studies may focus on more channels when the data is available. Secondly, this paper estimates public health through the health demand function, and adopts the mortality rate and life expectancy as the measurement indicators of public health. Future studies can use different public health measures based on the health supply function, which is conducive to enrich the contributions from novel perspectives.

## Data availability statement

The original contributions presented in the study are included in the article/Supplementary material, further inquiries can be directed to the corresponding author.

## Author contributions

ZZ: conceptualization, methodology, and formal analysis. MZ: data curation and writing—original draft. YZ: visualization and investigation. YF: writing—review and editing, supervision, and resources. All authors contributed to the article and approved the submitted version.

## References

[1] Gong P Liang S Carlton EJ Jiang Q Wu J Wang L. Urbanisation and health in China. Lancet. (2012) 379:843–52. 10.1016/S0140-6736(11)61878-3 22386037 PMC3733467

[2] Kabisch N van den Bosch M Lafortezza R. The health benefits of nature-based solutions to urbanization challenges for children and the elderly–a systematic review. Environ Res. (2017) 159:362–73. 10.1016/j.envres.2017.08.004 28843167

[3] Kegler SR Stone DM Holland KM. Trends in suicide by level of urbanization—United States, 1999–2015. Morbid Mortal Wkly Rep. (2017) 66:270. 10.15585/mmwr.mm6610a2 28301448 PMC5657870

[4] Li X Song J Lin T Dixon J Zhang G Ye H. Urbanization and health in China, thinking at the national, local and individual levels. Environ Health. (2016) 15:113–23. 10.1186/s12940-016-0104-5 26961780 PMC4895783

[5] McDade TW Adair LS. Defining the “urban” in urbanization and health: a factor analysis approach. Soc Sci Med. (2001) 53:55–70. 10.1016/S0277-9536(00)00313-0 11380161

[6] Chen H Liu Y Li Z Xue D. Urbanization, economic development and health: evidence from China's labor-force dynamic survey. Int J Equity Health. (2017) 16:1–8. 10.1186/s12939-017-0705-9 29187257 PMC5707809

[7] Goryakin Y. Suhrcke M. (2014). Economic development, urbanization, technological change and overweight: what do we learn from 244 demographic and health surveys? Econ Hum Biol. 14, 109–127. 10.1016/j.ehb.2013.11.003 24457038 PMC4330986

[8] Hou B Nazroo J Banks J Marshall A. Are cities good for health? A study of the impacts of planned urbanization in China. Int J Epidemiol. (2019) 48:1083–90. 10.1093/ije/dyz031 30887030

[9] Oster E. Unobservable selection and coefficient stability: theory and evidence. J Bus Econ Stat. (2019) 37:187–204. 10.1080/07350015.2016.1227711

[10] Lewbel A. Using heteroscedasticity to identify and estimate mismeasured and endogenous regressor models. J Bus Econ Stat. (2012) 30:67–80. 10.1080/07350015.2012.643126

[11] Deaton A. Income, health, and well-being around the world: evidence from the Gallup World Poll. J Econ Perspect. (2008) 22:53–72. 10.1257/jep.22.2.53 19436768 PMC2680297

[12] Turok I McGranahan G. Urbanization and economic growth: the arguments and evidence for Africa and Asia. Environ Urban. (2013) 25:465–82. 10.1177/0956247813490908

[13] Liu Y Xiao H Lv Y Zhang N. The effect of new-type urbanization on energy consumption in China: a spatial econometric analysis. J Clean Prod. (2017) 163:S299–305. 10.1016/j.jclepro.2015.10.044

[14] Chen J Wang L Li Y. Natural resources, urbanization and regional innovation capabilities. Resources Policy. (2020) 66:101643. 10.1016/j.resourpol.2020.101643

[15] Fan Y Fang C Zhang Q. Coupling coordinated development between social economy and ecological environment in Chinese provincial capital cities-assessment and policy implications. J Clean Prod. (2019) 229:289–98. 10.1016/j.jclepro.2019.05.027

[16] Ng BJ Zhou J Giannis A Chang VWC Wang JY. Environmental life cycle assessment of different domestic wastewater streams: policy effectiveness in a tropical urban environment. J Environ Manage. (2014) 140:60–8. 10.1016/j.jenvman.2014.01.052 24726966

[17] Qi F Abu-Rumman A Al Shraah A Muda I Huerta-Soto R Hai Yen TT. Moving a step closer towards environmental sustainability in Asian countries: focusing on real income, urbanization, transport infrastructure, and research and development. Econ Res. (2022) 1–20. 10.1080/1331677X.2022.2111317

[18] Gurjar BR Ravindra K Nagpure AS. Air pollution trends over Indian megacities and their local-to-global implications. Atmos Environ. (2016) 142:475–95. 10.1016/j.atmosenv.2016.06.030

[19] Seto KC Golden JS Alberti M Turner BL. Sustainability in an urbanizing planet. Proc Nat Acad Sci. (2017) 114:8935–8. 10.1073/pnas.1606037114 28784798 PMC5576777

[20] Han L Zhou W Li W Li L. Impact of urbanization level on urban air quality: a case of fine particles (PM[[sb]]2.5[[/s]]) in Chinese cities. Environ Pollut. (2014) 194:163–70. 10.1016/j.envpol.2014.07.022 25113968

[21] Satterthwaite D McGranahan G Tacoli C. Urbanization and its implications for food and farming. Philos Trans R Soc B Biol Sci. (2010) 365:2809–20. 10.1098/rstb.2010.0136 20713386 PMC2935117

[22] Dutton DJ Forest PG Kneebone RD Zwicker JD. Effect of provincial spending on social services and health care on health outcomes in Canada: an observational longitudinal study. CMAJ. (2018) 190:E66–71. 10.1503/cmaj.170132 29358200 PMC5780265

[23] Zhang Z Zhang G Li L. The spatial impact of atmospheric environmental policy on public health based on the mediation effect of air pollution in China. Environ Sci Pollut Res. (2022) 1–17. 10.1007/s11356-022-21501-6 35779217

[24] Contoyannis P Jones AM. Socio-economic status, health and lifestyle. J Health Econ. (2004) 23:965–95. 10.1016/j.jhealeco.2004.02.001 15353189

[25] Emanuel EJ Persad G Upshur R Thome B Parker M Glickman A. Fair allocation of scarce medical resources in the time of Covid-19. New Engl J Med. (2020) 382:2049–55. 10.1056/NEJMsb2005114 32202722

[26] Kavikondala S Stewart SM Ni MY Chan BH Lee PH Li KK. Structure and validity of Family Harmony Scale: an instrument for measuring harmony. Psychol Assess. (2016) 28:307. 10.1037/pas0000131 26146946

[27] Kolbe LJ. School health as a strategy to improve both public health and education. Annu Rev Public Health. (2019) 40:443–63. 10.1146/annurev-publhealth-040218-043727 30566386

[28] Nielsen TS Hansen KB. Do green areas affect health? Results from a Danish survey on the use of green areas and health indicators. Health Place. (2007) 13:839–50. 10.1016/j.healthplace.2007.02.001 17392016

[29] Ferkol T Schraufnagel D. The global burden of respiratory disease. Ann Am Thorac Soc. (2014) 11:404–6. 10.1513/AnnalsATS.201311-405PS 24673696

[30] Sidney S Quesenberry CP Jaffe MG Sorel M Nguyen-Huynh MN Kushi LH. Recent trends in cardiovascular mortality in the United States and public health goals. JAMA Cardiol. (2016) 1:594–9. 10.1001/jamacardio.2016.1326 27438477

[31] Crimmins EM Zhang Y Saito Y. Trends over 4 decades in disability-free life expectancy in the United States. Am J Public Health. (2016) 106:1287–93. 10.2105/AJPH.2016.303120 27077352 PMC4984740

[32] Thurber KA Thandrayen J Maddox R Barrett EM Walker J Priest N. Reflection on modern methods: statistical, policy and ethical implications of using age-standardized health indicators to quantify inequities. Int J Epidemiol. (2022) 51:324–33. 10.1093/ije/dyab132 34223891 PMC8855998

[33] Liang L Wang Z Li J. The effect of urbanization on environmental pollution in rapidly developing urban agglomerations. J Clean Prod. (2019) 237:117649. 10.1016/j.jclepro.2019.117649

[34] Qadeer A Saqib ZA Ajmal Z Xing C Khalil SK Usman M. Concentrations, pollution indices and health risk assessment of heavy metals in road dust from two urbanized cities of Pakistan: comparing two sampling methods for heavy metals concentration. Sustain Cities Soc. (2020) 53:101959. 10.1016/j.scs.2019.101959

[35] Liddle B Lung S. Might electricity consumption cause urbanization instead? Evidence from heterogeneous panel long-run causality tests. Global Environ Change. (2014) 24:42–51. 10.1016/j.gloenvcha.2013.11.013

[36] Lee KK Bing R Kiang J Bashir S Spath N Stelzle D. Adverse health effects associated with household air pollution: a systematic review, meta-analysis, and burden estimation study. Lancet Global Health. (2020) 8:e1427–34. 10.1016/S2214-109X(20)30343-0 33069303 PMC7564377

[37] Zhang S Wu Y Liu X Qian J Chen J Han L. Co-benefits of deep carbon reduction on air quality and health improvement in Sichuan Province of China. Environ Res Lett. (2021) 16:095011. 10.1088/1748-9326/ac1133

[38] Zhang Z Zhang G Su B. The spatial impacts of air pollution and socio-economic status on public health: empirical evidence from China. Socioecon Plann Sci. (2022) 83:101167. 10.1016/j.seps.2021.101167

[39] Xie M Liu X Yan W Li Y Liu X Zhang G. Carbon emission reduction pathways under carbon neutrality targets in Gansu province of China. Front Environ Sci. (2022) 2336. 10.3389/fenvs.2022.1042344

[40] Son JY Kim H Bell ML. Does urban land-use increase risk of asthma symptoms? Environ Res. (2015) 142:309–18. 10.1016/j.envres.2015.06.042 26188632

[41] Lambert KG Nelson RJ Jovanovic T Cerdá M. Brains in the city: neurobiological effects of urbanization. Neurosci Biobehav Rev. (2015) 58:107–22. 10.1016/j.neubiorev.2015.04.007 25936504 PMC4774049

[42] Hassell JM Begon M Ward MJ Fèvre EM. Urbanization and disease emergence: dynamics at the wildlife–livestock–human interface. Trends Ecol Evol. (2017) 32:55–67. 10.1016/j.tree.2016.09.012 28029378 PMC5214842

[43] Brenner N Ghosh S. Between the colossal and the catastrophic: planetary urbanization and the political ecologies of emergent infectious disease. Environ Plan A Econ Space. (2022) 54:0308518X221084313. 10.1177/0308518X221084313

[44] Patil RR. Urbanization as a determinant of health: a socioepidemiological perspective. Soc Work Public Health. (2014) 29:335–41. 10.1080/19371918.2013.821360 24871771

[45] Qin X Li L Hsieh CR. Too few doctors or too low wages? Labor supply of health care professionals in China. China Econ Rev. (2013) 24:150–64. 10.1016/j.chieco.2012.12.002

[46] Shen L Ren Y Xiong N Li H Chen Y. Why small towns can not share the benefits of urbanization in China? J Clean Prod. (2018) 174:728–38. 10.1016/j.jclepro.2017.10.150

[47] Wang X Chen J Burström B Burström K. Exploring pathways to outpatients' satisfaction with health care in Chinese public hospitals in urban and rural areas using patient-reported experiences. Int J Equity Health. (2019) 18:1–13. 10.1186/s12939-019-0932-3 30728005 PMC6366112

[48] Vlahov D Freudenberg N Proietti F Ompad D Quinn A Nandi V. Urban as a determinant of health. J Urban Health. (2007) 84:16–26. 10.1007/s11524-007-9169-3 17356903 PMC1891649

[49] Miao J Wu X. Urbanization, socioeconomic status and health disparity in China. Health Place. (2016) 42:87–95. 10.1016/j.healthplace.2016.09.008 27750075

[50] Lounkaew K. Explaining urban–rural differences in educational achievement in Thailand: evidence from PISA literacy data. Econ Educ Rev. (2013) 37:213–25. 10.1016/j.econedurev.2013.09.003

[51] Chauvin JP Glaeser E Ma Y Tobio K. What is different about urbanization in rich and poor countries? Cities in Brazil, China, India and the United States. J Urban Econ. (2017) 98:17–49. 10.1016/j.jue.2016.05.003

[52] Khullar D Chokshi DA. Health, income, & poverty: where we are & what could help. Health Aff. (2018) 10. 10.1377/hpb20180817.901935

[53] Grossman M. On the concept of health capital and the demand for health. J Polit Econ. (1972) 80:223–55. 10.1086/259880

[54] Bayati M Akbarian R Kavosi Z. Determinants of life expectancy in eastern mediterranean region: a health production function. Int J Health Policy Manage. (2013) 1:57. 10.15171/ijhpm.2013.09 24596837 PMC3937941

[55] Hartwig J Sturm JE. Testing the Grossman model of medical spending determinants with macroeconomic panel data. Eur J Health Econ. (2018) 19:1067–86. 10.1007/s10198-018-0958-2 29453763

[56] Jiang TB Deng ZW Zhi YP Cheng H Gao Q. The effect of urbanization on population health: evidence from China. Front Public Health. (2021) 2021:766. 10.3389/fpubh.2021.706982 34222193 PMC8242255

[57] Sosa-Rubí SG Galárraga O López-Ridaura R. Diabetes treatment and control: the effect of public health insurance for the poor in Mexico. Bull World Health Org. (2009) 87:512–9. 10.2471/BLT.08.053256 19649365 PMC2704037

[58] Biggs B King L Basu S Stuckler D. Is wealthier always healthier? The impact of national income level, inequality, and poverty on public health in Latin America. Soc Sci Med. (2010) 71:266–73. 10.1016/j.socscimed.2010.04.002 20471147

[59] Westoff CF. The recent fertility transition in Rwanda. Popul Dev Rev. (2013) 38:169–78. 10.1111/j.1728-4457.2013.00558.x

[60] Sisko AM Keehan SP Poisal JA Cuckler GA Smith SD Madison AJ. National health expenditure projections, 2018–27: economic and demographic trends drive spending and enrollment growth. Health Aff. (2019) 38:491–501. 10.1377/hlthaff.2018.05499 30785832

[61] Pfaller MA Diekema D. Epidemiology of invasive candidiasis: a persistent public health problem. Clin Microbiol Rev. (2007) 20:133–63. 10.1128/CMR.00029-06 17223626 PMC1797637

[62] Buxbaum JD Chernew ME Fendrick AM Cutler DM. Contributions of public health, pharmaceuticals, and other medical care to US life expectancy changes, 1990-2015. Health Aff. (2020) 39:1546–56. 10.1377/hlthaff.2020.00284 32897792

[63] DeFries RS Rudel T Uriarte M Hansen M. Deforestation driven by urban population growth and agricultural trade in the twenty-first century. Nat Geosci. (2010) 3:178–81. 10.1038/ngeo756

[64] Güneralp B Lwasa S Masundire H Parnell S Seto KC. Urbanization in Africa: challenges and opportunities for conservation. Environ Res Lett. (2017) 13:015002. 10.1088/1748-9326/aa94fe

[65] Maddala GS Wu S. A comparative study of unit root tests with panel data and a new simple test. Oxf Bull Econ Stat. (1999) 61:631–52. 10.1111/1468-0084.0610s1631

[66] Choi I. Unit root tests for panel data. J Int Money Finance. (2001) 20:249–72. 10.1016/S0261-5606(00)00048-6

[67] Arellano M Bover O. Another look at the instrumental variable estimation of error-components models. J Econom. (1995) 68:29–51. 10.1016/0304-4076(94)01642-D

[68] Ebeke CH Etoundi SMN. The effects of natural resources on urbanization, concentration, and living standards in Africa. World Dev. (2017) 96:408–17. 10.1016/j.worlddev.2017.03.02612267622

[69] Kakwani N. Performance in living standards: an international comparison. J Dev Econ. (1993) 41:307–36. 10.1016/0304-3878(93)90061-Q12733086

[70] Reinhart CM Rogoff KS. Recovery from financial crises: evidence from 100 episodes. Am Econ Rev. (2014) 104:50–5. 10.1257/aer.104.5.50

