# Supplementary material for: How does urbanization affect public health? New evidence from 175 countries worldwide
Source: Front Public Health. 2023 Jan 6;10:1096964. doi: 10.3389/fpubh.2022.1096964 (PMC9852986; doi:10.3389/fpubh.2022.1096964)
Supplement: Supplementary file 1 [file Data_Sheet_1.docx]

**Appendix**

**Appendix 1**

We adopt four methods for robustness checking. First, we replace the measurement indicators of public health and conduct a new regression to test whether the coefficient of the urbanization rate is significantly different from the original coefficient. Second, we use different urbanization rate measurement indicators for regression to test the robustness of the benchmark regression. Third, according to the regional division by World Bank, we divide the sample into six groups by region and run the group regressions. Finally, we add more control variables to ensure the reliability.

**Alternative measures of public health**

We use the under-five mortality rate (log*mort*) as a proxy variable for public health for robustness tests. The regression results are listed in Table A1. The coefficient of urbanization rate is -0.411 and is significant at the 5% level. This means that the urbanization rate has a significant negative impact on the under-five mortality rate. This is not different from our benchmark regression results.

**Table A1**

The results of urbanization rate and under-five mortality rate.

|  | (1) | (2) | (3) |
| --- | --- | --- | --- |
| VARIABLES | log*mort* | log*mortf* | log*mortm* |
| log*urban* | -0.411** | -0.444** | -0.384* |
|  | (-2.01) | (-2.15) | (-1.90) |
| log*gdp* | -0.396*** | -0.404*** | -0.388*** |
|  | (-6.20) | (-6.17) | (-6.18) |
| log*school* | 0.020 | 0.005 | 0.034 |
|  | (0.20) | (0.05) | (0.34) |
| log*fert* | -0.165 | -0.134 | -0.191* |
|  | (-1.49) | (-1.19) | (-1.74) |
| log*hexp* | 0.030 | 0.030 | 0.029 |
|  | (0.64) | (0.66) | (0.61) |
| *Constant*  *Country FE*  *Time FE* | Yes  Yes  Yes | Yes  Yes  Yes | Yes  Yes  Yes |
| *Observations* | 2,657 | 2,657 | 2,657 |
| *R^2^* | 0.806 | 0.805 | 0.807 |

**Alternative measures of urbanization**

Next, we replace the core explanatory variables for the robustness test. According to Liddle and Lung (2014), there is a significant correlation between the urbanization rate and the availability of electricity. Therefore, we use electricity availability (log*elec*) as a proxy variable for the urbanization rate. Table A2 reports the results of the regressions. From column (1) of Table A2, we can see that the availability of electricity significantly inhibits crude mortality. Specifically, a 1 percent increase in electricity availability is associated with a 0.223 percent decrease in crude mortality. As shown in columns (2) to (4) of Table A2, the regression results of available electricity and life expectancy are also as expected. This means that the results of the benchmark regression are reliable.

**Table A2**

The results of access to electricity and public health.

|  | (1) | (2) | (3) | (4) |
| --- | --- | --- | --- | --- |
| VARIABLES | log*death* | log*life* | log*lifef* | log*lifem* |
| log*elec* | -0.223*** | 0.059*** | 0.061*** | 0.056*** |
|  | (-3.90) | (4.07) | (4.19) | (3.86) |
| log*gdp* | -0.029 | 0.033*** | 0.035*** | 0.031*** |
|  | (-0.68) | (3.45) | (3.54) | (3.25) |
| log*school* | -0.278*** | 0.060*** | 0.060*** | 0.060*** |
|  | (-3.69) | (2.97) | (3.08) | (2.85) |
| log*fert* | 0.164** | -0.021 | -0.033** | -0.009 |
|  | (2.03) | (-1.25) | (-2.01) | (-0.52) |
| log*hexp* | 0.048 | -0.012 | -0.012 | -0.012 |
|  | (1.19) | (-1.26) | (-1.29) | (-1.20) |
| *Constant* | Yes | Yes | Yes | Yes |
| *Country FE*  *Time FE* | Yes  Yes | Yes  Yes | Yes  Yes | Yes  Yes |
| *Observations* | 2,657 | 2,656 | 2,656 | 2,656 |
| *R^2^* | 0.491 | 0.697 | 0.687 | 0.702 |

**Dividing into groups**

Because of the different levels of economic development and natural environment in different regions of the world, we cannot conclude that the increase of urbanization rate will promote public health by studying the whole sample. Thus, according to regional division by World Bank, countries are clustered into six groups: East Asia and Pacific (EAP), European and Central Africa (ECA), Latin America and the Caribbean (LAC), Middle East and North Africa (MENA), Sub-Saharan Africa and other regions (the region not included in the five groups). We regress each of the six samples. Tables A3a to A3f list the regression results. According to the results of the table, only the coefficient of urbanization in Latin America and the Caribbean has a positive impact on crude mortality and a negative impact on life expectancy. In the remaining five subsamples, the coefficient of urbanization rate is in line with expectations, and is basically significant at the 1 percent level.

**Table A3a**

Urbanization rate and public health (other regions), two-way fixed effect estimation.

|  | (1) | (2) | (3) | (4) |
| --- | --- | --- | --- | --- |
| VARIABLES | log*death* | log*life* | log*lifef* | log*lifem* |
| log*urban* | -0.008 | 0.074*** | 0.090*** | 0.068** |
|  | (-0.05) | (3.26) | (4.75) | (2.42) |
| log*gdp* | -0.054 | 0.047*** | 0.046*** | 0.044*** |
|  | (-1.27) | (9.09) | (9.50) | (7.70) |
| log*school* | 0.226** | -0.023** | -0.021** | -0.022* |
|  | (2.48) | (-2.12) | (-2.16) | (-1.79) |
| log*fert* | 0.745*** | -0.102*** | -0.109*** | -0.096*** |
|  | (5.74) | (-5.71) | (-6.84) | (-4.56) |
| log*hexp* | 0.147*** | -0.012*** | -0.017*** | -0.009* |
|  | (4.66) | (-2.92) | (-4.58) | (-1.85) |
| *Constant* | Yes | Yes | Yes | Yes |
| *Country FE*  *Time FE* | Yes  Yes | Yes  Yes | Yes  Yes | Yes  Yes |
| *Observations* | 160 | 160 | 160 | 160 |
| *R^2^* | 0.976 | 0.997 | 0.998 | 0.996 |

**Table A3b**

Urbanization rate and public health (East Asia and Pacific region), two-way fixed effect estimation.

|  | (1) | (2) | (3) | (4) |
| --- | --- | --- | --- | --- |
| VARIABLES | log*death* | log*life* | log*lifef* | log*lifem* |
| log*urban* | -0.270*** | 0.087*** | 0.097*** | 0.078*** |
|  | (-3.01) | (5.06) | (5.79) | (4.32) |
| log*gdp* | -0.070* | 0.027*** | 0.024*** | 0.029*** |
|  | (-1.83) | (3.41) | (3.15) | (3.58) |
| log*school* | -0.341*** | 0.083*** | 0.082*** | 0.084*** |
|  | (-7.60) | (5.87) | (5.91) | (5.83) |
| log*fert* | 0.334*** | -0.060*** | -0.056*** | -0.065*** |
|  | (4.89) | (-4.22) | (-3.81) | (-4.66) |
| log*hexp* | -0.020 | -0.003 | -0.002 | -0.004 |
|  | (-0.80) | (-0.85) | (-0.60) | (-1.05) |
| *Constant* | Yes | Yes | Yes | Yes |
| *Country FE* | Yes | Yes | Yes | Yes |
| *Time FE* | Yes | Yes | Yes | Yes |
| *Observations* | 375 | 375 | 375 | 375 |
| *R^2^* | 0.938 | 0.986 | 0.987 | 0.985 |

**Table A3c**

Urbanization rate and public health (European and Central Africa region), two-way fixed effect estimation.

|  | (1) | (2) | (3) | (4) |
| --- | --- | --- | --- | --- |
| VARIABLES | log*death* | log*life* | log*lifef* | log*lifem* |
| log*urban* | -0.061 | 0.035** | 0.013 | 0.057** |
|  | (-0.57) | (2.06) | (0.90) | (2.53) |
| log*gdp* | -0.003 | 0.021*** | 0.023*** | 0.019*** |
|  | (-0.11) | (6.80) | (7.45) | (5.46) |
| log*school* | -0.290*** | -0.007 | -0.017** | 0.001 |
|  | (-4.58) | (-0.67) | (-2.47) | (0.08) |
| log*fert* | -0.015 | 0.015** | 0.003 | 0.027*** |
|  | (-0.34) | (2.24) | (0.50) | (3.44) |
| log*hexp* | 0.060*** | -0.003 | -0.002 | -0.004 |
|  | (3.02) | (-1.13) | (-0.67) | (-1.18) |
| *Constant* | Yes | Yes | Yes | Yes |
| *Country FE* | Yes | Yes | Yes | Yes |
| *Time FE* | Yes | Yes | Yes | Yes |
| *Observations* | 796 | 796 | 796 | 796 |
| *R^2^* | 0.965 | 0.985 | 0.985 | 0.984 |

**Table A3d**

Urbanization rate and public health (Latin America and the Caribbean region), two-way fixed effect estimation.

|  | (1) | (2) | (3) | (4) |
| --- | --- | --- | --- | --- |
| VARIABLES | log*death* | log*life* | log*lifef* | log*lifem* |
| log*urban* | 0.231** | -0.027* | -0.019 | -0.034** |
|  | (2.27) | (-1.77) | (-1.43) | (-2.06) |
| log*gdp* | -0.181*** | 0.045*** | 0.050*** | 0.038*** |
|  | (-7.47) | (8.77) | (10.33) | (6.59) |
| log*school* | 0.329*** | -0.056*** | -0.061*** | -0.050*** |
|  | (4.40) | (-4.20) | (-4.33) | (-3.96) |
| log*fert* | 0.747*** | -0.127*** | -0.123*** | -0.129*** |
|  | (9.36) | (-9.71) | (-9.33) | (-9.39) |
| log*hexp* | 0.098*** | -0.012*** | -0.013*** | -0.009** |
|  | (4.39) | (-3.26) | (-3.40) | (-2.42) |
| *Constant* | Yes | Yes | Yes | Yes |
| *Country FE* | Yes | Yes | Yes | Yes |
| *Time FE* | Yes | Yes | Yes | Yes |
| *Observations* | 438 | 438 | 438 | 438 |
| *R^2^* | 0.967 | 0.974 | 0.970 | 0.977 |

**Table A3e**

Urbanization rate and public health (Middle East and North Africa region), two-way fixed effect estimation.

|  | (1) | (2) | (3) | (4) |
| --- | --- | --- | --- | --- |
| VARIABLES | log*death* | log*life* | log*lifef* | log*lifem* |
| log*urban* | -0.064 | 0.180*** | 0.187*** | 0.178*** |
|  | (-0.33) | (8.94) | (9.32) | (8.36) |
| log*gdp* | -0.019 | 0.024*** | 0.022*** | 0.024*** |
|  | (-0.33) | (3.38) | (3.12) | (3.36) |
| log*school* | -0.405*** | 0.060*** | 0.056*** | 0.070*** |
|  | (-5.01) | (5.80) | (5.67) | (6.38) |
| log*fert* | 0.439*** | 0.006 | -0.004 | 0.015** |
|  | (5.46) | (0.97) | (-0.63) | (2.41) |
| log*hexp* | 0.135*** | 0.001 | 0.002 | 0.000 |
|  | (3.48) | (0.29) | (0.56) | (0.15) |
| *Constant* | Yes | Yes | Yes | Yes |
| *Country FE* | Yes | Yes | Yes | Yes |
| *Time FE* | Yes | Yes | Yes | Yes |
| *Observations* | 239 | 239 | 239 | 239 |
| *R^2^* | 0.992 | 0.988 | 0.987 | 0.988 |

**Table A3f**

Urbanization rate and public health (Sub-Saharan Africa), two-way fixed effect estimation.

|  | (1) | (2) | (3) | (4) |
| --- | --- | --- | --- | --- |
| VARIABLES | log*death* | log*life* | log*lifef* | log*lifem* |
| log*urban* | -0.571*** | 0.108*** | 0.086** | 0.128*** |
|  | (-4.01) | (2.63) | (2.02) | (3.23) |
| log*gdp* | -0.077* | 0.059*** | 0.059*** | 0.059*** |
|  | (-1.79) | (4.06) | (3.93) | (4.12) |
| log*school* | -0.162*** | 0.013 | 0.019 | 0.008 |
|  | (-3.24) | (0.81) | (1.21) | (0.53) |
| log*fert* | 0.731*** | -0.196*** | -0.190*** | -0.202*** |
|  | (4.70) | (-4.24) | (-3.97) | (-4.52) |
| log*hexp* | 0.073*** | -0.029*** | -0.029*** | -0.028*** |
|  | (2.68) | (-3.05) | (-3.14) | (-2.93) |
| *Constant* | Yes | Yes | Yes | Yes |
| *Country FE* | Yes | Yes | Yes | Yes |
| *Time FE* | Yes | Yes | Yes | Yes |
| *Observations* | 642 | 642 | 642 | 642 |
| *R^2^* | 0.917 | 0.940 | 0.939 | 0.942 |

**Adding more controls**

At the end of the robustness check, we add more control variables to the model. We first add trade openness (log*trade*) to the benchmark regression model. According to the study of Shaffer et al. (1), trade openness will impact public health. Specifically, trade openness contributes to national economic development, promoting the region's public health. In addition, Demir et al. (2) also showed that positive trade opening shock has a negative impact on public health expenditure, while negative trade opening shock has a positive impact on public health expenditure. Second, we add foreign direct investment (log*fdi*) as a control variable to the model, as suggested by Burns (3). FDI creates more employment opportunities for local people and contributes to the development of the local economy. More jobs and economic growth may lead to better public health. Therefore, FDI will also promote public health.

**Table A4**

The results of adding more controls.

|  | (1) | (2) | (3) | (4) |
| --- | --- | --- | --- | --- |
| VARIABLES | log*death* | log*life* | log*lifef* | log*lifem* |
| log*urban* | -0.664*** | 0.195*** | 0.196*** | 0.193*** |
|  | (-3.04) | (3.50) | (3.60) | (3.38) |
| log*gdp* | -0.024 | 0.030** | 0.032** | 0.028** |
|  | (-0.43) | (2.31) | (2.38) | (2.19) |
| log*school* | -0.382*** | 0.077*** | 0.079*** | 0.075*** |
|  | (-4.35) | (3.19) | (3.26) | (3.08) |
| log*fert* | 0.216** | -0.034 | -0.047** | -0.020 |
|  | (2.23) | (-1.46) | (-2.04) | (-0.86) |
| log*hexp* | 0.056 | -0.016 | -0.016 | -0.016 |
|  | (1.29) | (-1.44) | (-1.41) | (-1.43) |
| log*trade* | 0.009 | 0.000 | 0.000 | 0.001 |
|  | (0.65) | (0.11) | (0.01) | (0.20) |
| log*fdi* | -0.000 | 0.000 | 0.000 | -0.000 |
|  | (-0.03) | (0.04) | (0.26) | (-0.19) |
| *Constant* | Yes | Yes | Yes | Yes |
| *Country FE* | Yes | Yes | Yes | Yes |
| *Time FE* | Yes | Yes | Yes | Yes |
| *Observations* | 2,400 | 2,400 | 2,400 | 2,400 |
| *R^2^* | 0.425 | 0.664 | 0.649 | 0.674 |

Table A4 reports the regression results after adding control variables. As presented in Table A4, the coefficients of the urbanization rate are consistent with those above, and the benchmark regression results are still robust. Trade openness and FDI have no significant impact on public health, which may be caused by the fact that these two variables have a minor impact on the public health level of each country.

**Appendix 2**

**Table A5**

List of countries.

| Country | World bank country code |  | Country | World bank country code |
| --- | --- | --- | --- | --- |
| Afghanistan | AFG |  | Albania | ALB |
| Algeria | DZA |  | Angola | AGO |
| Antigua and Barbuda | ATG |  | Argentina | ARG |
| Armenia | ARM |  | Australia | AUS |
| Austria | AUT |  | Azerbaijan | AZE |
| Bahamas, The | BHS |  | Bahrain | BHR |
| Bangladesh | BGD |  | Barbados | BRB |
| Belarus | BLR |  | Belgium | BEL |
| Belize | BLZ |  | Benin | BEN |
| Bhutan | BTN |  | Bolivia | BOL |
| Botswana | BWA |  | Brazil | BRA |
| Brunei Darussalam | BRN |  | Bulgaria | BGR |
| Burkina Faso | BFA |  | Burundi | BDI |
| Cabo Verde | CPV |  | Cambodia | KHM |
| Cameroon | CMR |  | Canada | CAN |
| Central African Republic | CAF |  | Chad | TCD |
| Chile | CHL |  | China | CHN |
| Colombia | COL |  | Comoros | COM |
| Congo, Dem. Rep. | COD |  | Congo, Rep. | COG |
| Costa Rica | CRI |  | Cote d'Ivoire | CIV |
| Croatia | HRV |  | Cyprus | CYP |
| Czech Republic | CZE |  | Denmark | DNK |
| Djibouti | DJI |  | Dominica | DMA |
| Dominican Republic | DOM |  | Ecuador | ECU |
| Egypt, Arab Rep. | EGY |  | El Salvador | SLV |
| Equatorial Guinea | GNQ |  | Estonia | EST |
| Eswatini | SWZ |  | Ethiopia | ETH |
| Fiji | FJI |  | Finland | FIN |
| France | FRA |  | Gabon | GAB |
| Gambia | GMB |  | Georgia | GEO |
| Germany | DEU |  | Ghana | GHA |
| Greece | GRC |  | Grenada | GRD |
| Guatemala | GTM |  | Guinea | GIN |
| Guinea-Bissau | GNB |  | Guyana | GUY |
| Honduras | HND |  | Hungary | HUN |
| Iceland | ISL |  | India | IND |
| Indonesia | IDN |  | Iran, Islamic Rep. | IRN |
| Iraq | IRQ |  | Ireland | IRL |
| Israel | ISR |  | Italy | ITA |
| Jamaica | JAM |  | Jordan | JOR |
| Kazakhstan | KAZ |  | Kenya | KEN |
| Kiribati | KIR |  | Korea, Rep. | KOR |
| Kuwait | KWT |  | Kyrgyz Republic | KGZ |
| Lao PDR | LAO |  | Latvia | LVA |
| Lesotho | LSO |  | Liberia | LBR |
| Libya | LBY |  | Lithuania | LTU |
| Luxembourg | LUX |  | Madagascar | MDG |
| Malawi | MWI |  | Malaysia | MYS |
| Maldives | MDV |  | Mali | MLI |
| Malta | MLT |  | Marshall Islands | MHL |
| Mauritania | MRT |  | Mauritius | MUS |
| Mexico | MEX |  | Micronesia, Fed. Sts. | FSM |
| Moldova | MDA |  | Mongolia | MNG |
| Montenegro | MNE |  | Morocco | MAR |
| Mozambique | MOZ |  | Myanmar | MMR |
| Namibia | NAM |  | Nepal | NPL |
| Netherlands | NLD |  | New Zealand | NZL |
| Nicaragua | NIC |  | Niger | NER |
| Nigeria | NGA |  | North Macedonia | MKD |
| Norway | NOR |  | Oman | OMN |
| Pakistan | PAK |  | Palau | PLW |
| Panama | PAN |  | Papua New Guinea | PNG |
| Paraguay | PRY |  | Peru | PER |
| Philippines | PHL |  | Poland | POL |
| Portugal | PRT |  | Qatar | QAT |
| Romania | ROU |  | Russian Federation | RUS |
| Rwanda | RWA |  | Samoa | WSM |
| San Marino | SMR |  | Sao Tome and Principe | STP |
| Saudi Arabia | SAU |  | Senegal | SEN |
| Serbia | SRB |  | Seychelles | SYC |
| Sierra Leone | SLE |  | Singapore | SGP |
| Slovak Republic | SVK |  | Slovenia | SVN |
| Solomon Islands | SLB |  | South Africa | ZAF |
| Spain | ESP |  | Sri Lanka | LKA |
| St. Kitts and Nevis | KNA |  | St. Lucia | LCA |
| St. Vincent and the Grenadines | VCT |  | Sudan | SDN |
| Suriname | SUR |  | Sweden | SWE |
| Switzerland | CHE |  | Tajikistan | TJK |
| Tanzania | TZA |  | Thailand | THA |
| Timor-Leste | TLS |  | Togo | TGO |
| Tonga | TON |  | Trinidad and Tobago | TTO |
| Tunisia | TUN |  | Turkey | TUR |
| Uganda | UGA |  | Ukraine | UKR |
| United Arab Emirates | ARE |  | United Kingdom | GBR |
| United States | USA |  | Uruguay | URY |
| Uzbekistan | UZB |  | Vanuatu | VUT |
| Vietnam | VNM |  | Zambia | ZMB |
| Zimbabwe | ZWE |  |  |  |

## References

1. Shaffer ER, Waitzkin H, Brenner J, Jasso-Aguilar R. Global trade and public health. *Am J Public Health*. (2005) 95:23–34. doi: 10.2105/AJPH.2004.038091

2. Demir S, Demir H, Karaduman C, Cetin M. Environmental quality and health expenditures efficiency in Türkiye: the role of natural resources. *Environ Sci Pollut Res*. (2022) 1–16. doi: 10.1007/s11356-022-23187-2

3. Burns DK, Jones AP, Goryakin Y, Suhrcke M. Is foreign direct investment good for health in low and middle income countries? An instrumental variable approach. *Soc Sci Med*. (2017) 181:74–82. doi: 10.1016/j.socscimed.2017.03.054
